# Does Less Optimal Nonverbal Communication with Peers Predict the Development of Depression in Adolescent Boys and Girls?

**DOI:** 10.1007/s10802-019-00517-6

**Published:** 2019-02-09

**Authors:** Yolanda van Beek, Anne Berg

**Affiliations:** Department of Developmental Psychology, Utrecht University, Heidelberglaan 1, 3584 CS Utrecht, the Netherlands

**Keywords:** Depressive symptoms, Nonverbal behavior, Adolescence, Gender differences

## Abstract

A Social Skills Deficit Model for depression in adolescence was tested, proposing that less optimal nonverbal behavior elicits negative reactions in peer partners, which in turn result in depressive symptoms. Adolescents (12–17 years of age) participated in videotaped same-sex interactions. Several positive and negative nonverbal behaviors were coded. Two analyses were conducted using longitudinal data collected in four waves. First, the predictive role of nonverbal communication for depressive symptoms was tested in a normative sample of 170 adolescent dyads without (mild) depression at wave 1 (48% girls). Second, in a subsample of 31 adolescents who developed (sub)clinical depression in wave 2–4, behaviors during peer interactions prior to the development of depression were compared with behaviors of 31 matched controls that did not show mild depression in any wave (55% girls). Only gazing behavior showed the expected relationships. In girls, less gazing in targets was related to less gazing in peers, and if this response occurred, it subsequently predicted later depressive symptoms of targets. The importance of gazing behavior was confirmed in the (sub)clinical sample where girls, prior to increases in depressive symptoms, gazed less and boys gazed more as compared to controls. Interaction partners of these girls and boys also responded with less gazing. The findings indicate that nonverbal social skills are related to the development of depression in youth, particularly in girls.

It is well known that depression in childhood and adolescence is often preceded by problems in social relationships, such as low popularity, lower social support, rejection or victimization by peers, and negative experiences in romantic relationships (Kupersmidt and Patterson 1991; La Greca and Harrison 2005; Nolan et al. 2003; Stice et al. 2004; Zwierzynska et al. 2013). Interpersonal stress, elicited by problems in peer relations, seems more strongly related to depression in girls than boys (Conley et al. 2012; Shih et al. 2006). But what causes these relationship problems? The Social Skills Deficit Model (SSDM) (Coyne 1976a, b; Segrin 2000) suggests that persons that develop depression possess less optimal social skills, that elicit negative feedback of others, which in turn results in negative self-images and depression. Evidence for this theory comes from questionnaire and experimental studies showing that lower social competence in adolescence indeed predict depressive symptoms (Engels et al. 2001; Segrin and Flora 2000), and studies showing that persons with depression are evaluated less positively by themselves and others (Hammen and Peters 1978; Segrin 2000). However, it is still not well understood what exactly goes awry in peer communication in terms of behavior, nor if the same deficits play a role in boys and girls. To find out which behaviors should be addressed in interventions, it is essential to study actual behavior. Therefore, the present observation study attempts to find out which behaviors of adolescents relate to negative responses from peers, that subsequently predict depression. Specific attention is given to possible gender differences in these behavioral predictors.

The SSDM for the development of depression seems particularly relevant for adolescent girls. Girls spend more time with peers (Johnson 2004), female friendships become characterized by more intimacy (Bank and Hansford 2000), and the desire for close relationships is stronger in adolescent girls than boys (Rose and Rudolph 2006). Yet, little is known about which behavioral patterns result in relationship problems and subsequent depression. The present study focusses on the possible role of nonverbal behavior, because nonverbal behaviors play an important role in developing relationships (DePaulo 1992). Moreover, nonverbal behavior shows a different development in boys and girls. These differences (in part) develop through socialization processes. In general, women are expected to be more caring, friendlier and other-oriented (Hall 1984; Plant et al. 2000), whereas negative behaviors are less accepted than in men (DePaulo 1992; Wallbott 1988). Although these display rules are mostly implicit and largely unconscious (DePaulo 1992), they do result in increasingly different nonverbal behaviors in boys and girls. An earlier observation study in adolescence showed that this development is still ongoing during adolescence (van Beek et al. 2006; van Beek and Berg 2015). From early to middle adolescence gazing and smiling increased and negative expressions decreased in girls’ dyads leading to larger differences with boys’ dyads as a result. Maybe such behaviors become increasingly important for girls, given that their friendships focus more on intimacy which requires a higher level of positive and ‘other-oriented’ behaviors? Therefore, the present study investigates whether girls that display less of these desired nonverbal behaviors receive more negative responses from same-sex peers, that may eventually lead to depression.

## Previous Research

Previous observation studies provide some evidence that persons with depression show deficits in nonverbal behaviors. Adults with depression showed less facial expressivity, smiling, gesturing, yes-nodding and gazing at interaction partners than controls without depression (Gaebel and Wölwer 2004; Segrin and Abramson 1994; Troisi and Moles 1999). Also, children with depression were found to smile less (Kazdin et al. 1985) and to experience more aggressive peer interactions (Altman and Gotlib 1988). Finally, adolescent girls with mild depression gazed less at their interaction partner while listening (van Beek et al. 2006) and showed more negative facial expressions and more body cues indicating discomfort or boredom than healthy control girls (van Beek and Berg 2015).

The SSDM further hypothesizes that the relation between social skill deficits and depression is mediated by negative reactions of others. Using questionnaires, a strong link between perceived social skills (aggression, withdrawal and prosocial behavior) and dislike by peers has been shown in pre-adolescents, where peer acceptance was a mediator between dislike and depression (Zimmer-Gembeck et al. 2007). However, it is not clear from this study how adolescents who later develop depression perceive that they are disliked. Probably, behavioral responses towards these adolescents are less positive. Observational studies of specific partners’ behaviors in response to persons with depression are scarce but did show that partners smile less at young adults with depression (Gotlib and Robinson 1982) and smile less and display more negative facial expressions towards adolescents with (mild) depression (van Beek et al. 2006; van Beek and Berg 2015).

## The Present Study

Previous studies thus provided some indications that deficits in nonverbal behavior are related to depressive symptoms, and that adolescents who already developed (mild) depression receive fewer positive responses from peers. However, it has not yet been shown that such behavioral patterns exist prior to the development of depression, nor that negative behavioral responses of peers mediate the link between social skills and depression. Therefore, to test the proposed SSDM, the present study investigates the suggested longitudinal link between social behaviors, peer responses and depressive symptoms. Hypotheses are that less optimal behaviors of target adolescents are related to more negative partner behaviors, and that this partner behavior mediates the relation between target behavior and later depressive symptoms.

To suggested model is tested for boys and girls. It is expected that strongest effects are found in girls, because of the higher importance of close peer relationships for well-being in girls (Rose and Rudolph 2006). Also, different behavioral levels may result in negative responses in boys and girls, due to (largely unconscious) differences in expectations. Although the meaning of nonverbal behaviors is similar in boys and girls, in girls more positive and fewer negative behaviors are expected than in boys. Gazing and smiling are considered as positive behaviors in both sexes but showing less of these behaviors is more acceptable in boys than in girls. And negative behaviors are more common and accepted in boys than in girls (Hall 1984). Peers may thus consider such behaviors as less important for relationship quality than in girls and therefore may not show a negative response. Nevertheless, it could be that also in boys more extreme levels of negative behaviors or lack of positive behaviors result in negative peer responses.

The proposed SSDM was tested in a normative school population using a four-wave longitudinal design, where behaviors of adolescents and same-sex peers without (mild) depression were observed to predict depressive symptoms in a later wave. Because a study using a normative sample primarily predicts variation in depressive symptoms in the normal range, also a small subsample was selected that developed (sub)clinical levels of depression at wave 2, 3 or 4. It was expected that the behavioral characteristics (of both target and peer partner) that predict later depressive symptoms in the normative sample, would also occur more often in the (sub)clinical group prior to the development of depressive symptoms, as compared to a matched control group that never developed (mild) depression.

## Methods

### Participants

Participants were selected from a larger sample of 606 participants recruited from two Dutch secondary schools, of which data were collected in four waves with on average 9 months apart. The study was approved by the medical ethical committee of the Utrecht University and confidentiality of collected data was ensured. Participants, their parents and the schools were informed about the global goals of the study. Informed consent^1^ was obtained from all individual participants included in the study. After approval, participants could stop participating at any time.

#### Sample of predictive analyses

To test the hypothesized longitudinal link between nonverbal communication and later depression scores, videotaped conversations of 170 adolescent dyads were selected from wave 1 (48% girls, *M*_*initial depression*_ = 3.76, *SD*_*initial depression*_ = 3.05), aged between 12 and 17 at wave 1 (*M*_*age*_ = 14.60, *SD*_*age*_ = 1.01). Only conversations were selected from an earlier sample (Van Beek et al. 2006), if both partners had a score below 13 on the Children’s Depression Inventory (CDI), based on Kovačs’ (1992) and Roelofs et al. (2010) cut-off value for mild depression. The conversation partners were matched on age and sex. If later depression scores were available for both partners in a dyad, both were entered in the analyses as a target (and the other as the partner).

#### (Sub)clinical sample for retrospective analyses

To study the clinical relevance of the findings of the predictive analyses, it was examined whether the same deficits in nonverbal behavior were found in a (sub)clinical subsample, prior to the development of (mild) depression. Therefore, 31 adolescents, who developed (sub)clinical levels of depression at a later wave were selected; 20 from the predictive sample described above, 6 from a later observation study using another selection from the same larger school population, and 5 participants who were included at wave 2 and developed mild depression in a later wave. To maximize the number of participants (specifically boys), also 5 adolescents with a score of 11 or 12 (>75th percentile) in a later wave, were selected, but only when they showed an increase in CDI score of at least four points.^2^ The mean depression score at the later wave was well above the more common cut-off of 13 for mild depression (*M* = 18.83, *SD* = 8.39). This (sub)clinical group of adolescents were compared to 31 controls, who did not develop (mild) depression (CDI ≤ 9 in all waves). The two groups were matched on age and gender, and had similar initial depression scores, *F*(1, 60) = 0.63, *p* = .432 (*M*_*to-be-depressed*_ = 5.18, *SD*_*to-be-depressed*_ = 2.59, *M*_*control*_ = 4.68, *SD*_*control*_ = 2.41). The partners in conversation did not show (mild) depression (CDI ≤ 9) and were matched on age and gender. The total subsample thus consisted of 62 adolescent dyads (55% girls, *M*_*age*_ = 14.69, *SD*_*age*_ = 1.00).

### Measures

#### Depressive Symptoms

Depressive symptoms were measured at each wave using a Dutch version (van Beek et al. 2012) of the Children’s Depression Inventory (CDI) (Kovačs 1992). This self-report questionnaire consists of 28 items where participants should indicate out of 3 descriptions (0 = not depressed, 1 = slightly/mildly depressed, and 2 = more clearly depressed), which description applied to them best. Reliability of this scale was good (Cronbach’s alpha = .81, van Beek et al. 2012).

### Observations

To examine nonverbal behavior of targets and partners, adolescents participated in 5-minute semi-structured conversations with a peer of the same sex and age that were recorded and coded. During these conversations participants discussed a social dilemma concerning relationships with peers (e.g., “*what would you do if you saw your close friend steal something from a store?*”) until they agreed upon the best solution. If the adolescents agreed on a solution before the 5 minutes elapsed, the couple was asked to make up a top-5 ranking (e.g., about the most popular teachers) until the 5 minutes elapsed. Instruction periods or short disruptions (e.g., responses to outside occurrences/sounds or questions asked to the experimenter) were not coded to ensure that only real interaction between participants was included. Interaction partners might know each other, however, were not friends, to avoid that differences in behavior were caused by variation in relatedness. To this end, we selected dyads consisting of participants from different classes and prior to recording we asked whether they were friends. If so, dyads were reorganized. Participants were unaware that their nonverbal behavior was observed.

#### Other-Oriented (Positive) Nonverbal Behavior

Gazing, talking, backchanneling and smiling of targets and interaction partners were coded one by one using Observer XT (Version 11), resulting in the following variables:

- *Gazing while talking*: duration of directing the eyes toward the face of the partner as a percentage of talking time.
- *Gazing while listening*: duration of directing the eyes toward the face of the partner as a percentage of talking time of the interaction partner.
- *Backchanneling*: frequency of listener’s behaviors, such as nodding, shaking or vocal utterances, like ‘yes’, ‘hmm-hmm’, that indicate interest or that confirm the partner’s speech, without interrupting him/her.
- *Smiling*: frequency per minute.

#### Negative Behaviors

Negative behavior was measured by two variables. ‘Negative facial expression’ was measured by coding facial expressions signaling negativity, rejection and/or contempt, such as frowning, yawning and a blank disinterested face, on a 5-point scale ranging from (almost) not occurring to occurring relatively often. The second variable, ‘subtle displeasure cues’ consisted of other signs of disinterest, boredom, and discomfort that were measured by coding movements and body postures mentioned in literature on behavioral observations in adults with depression (Bouhuys and Albersnagel 1992; Bouhuys and van den Hoofdakker 1991, 1993; Gotlib and Robinson 1982; Troisi and Moles 1999) and general literature regarding such negative behaviors in adults (Vrugt 1983) or children (Dumas et al. 1994). Each of the following cues was scored on a 5-point scale ranging from (almost) not occurring to occurring relatively often. As each signal only rarely occurred, they were summed into a total score ‘subtle displeasure cues’.

- *Facial restlessness*: e.g., rubbing lips, biting on lips or cheeks.
- *Tensed body position*: e.g., stiff posture, firmly closed arms, chin on chest.
- *Nervous body touching*: e.g., scratching, fidgeting with hair or clothes.
- *Cues of boredom/disinterest in movements*: e.g., explicitly chewing gum, drumming fingers.
- *Cues of boredom/disinterest in body posture*: e.g., slumped or slightly turned away position.
- *Lack of variation in intonation*: i.e., monotonous speech.

Nervous body touching and cues of boredom/disinterest in movements were coded simultaneously, as were cues of boredom/disinterest in body posture and monotonous speech. The other scales were coded one by one.

#### Reliability and Validity of Nonverbal Behaviors

Raters (20 graduate students) that coded the recordings were unaware of participants’ depression scores. They were usually trained for 1 or 2 ‘other-oriented’ behaviors or for 2 to 3 negative scales. Reliability was first tested on the same 20 ‘golden standard’ video recordings that were coded by the first author until they reached a Cohen’s Kappa of at least 0.70 (allowing for a difference in timing of 1 s for the other-oriented behaviors). Secondly, interrater reliability between experienced student raters and new raters, using more recently coded observations, was calculated by double coding at least 10 video recordings. Inter-rater reliability of all coded behaviors and scales was good, with Pearson’s *r* above .80 (range .81–.98, mean 0.90) and Kappa above .60 (range .61–.92, mean .74).

The validity of the behaviors, i.e., whether the selected behaviors correlated with the perceived quality of the conversation, was also examined. To this end, 171 participants of the larger population from wave 1 (including participants with higher depression scores) were asked to indicate on a 10-point scale how satisfied they were with the conversation, by answering four questions immediately after the recording: ‘How satisfied are you with the result of the discussion?’, ‘How much did you like the discussion with this person?’, ‘How much do you think the other liked the discussion with you?’, and ‘How well did you manage to come to an agreement?’. All other-oriented behaviors, except backchanneling, correlated significantly positive with the sum of own and/or the sum of partner satisfaction questions, with *r* ranging from .16 to .23, *p* < .05, for partner satisfaction, and from .27 to .32, *p* < .05, for own satisfaction. Negative behaviors correlated significantly negative with own and/or partner satisfaction, with *r* ranging from −.13 to −.24, *p* < .05, with partner satisfaction, and − .27 (only negative face), *p* < .05, with own satisfaction. Other-oriented behaviors were thus valued as positive and negative behaviors as negative.

### Analyses

#### Predictive Analyses

To examine the hypothesized relations between target adolescents’ behavior, partners’ nonverbal responses and targets’ depressive symptoms, path analyses using Structural Equation Modelling were executed in M*plus* Version 7.2 (Muthén and Muthén 2012).

As a first step, to deal with the large number of parameters in relation to the sample size, preliminary analyses on the observation data were performed to reduce the number of relations in the model. Backwards selection of significant correlations and regression paths was used in M*plus* to end up with only significant within- and between-person (i.e., target to partner behavior) relations. Only significant (*p* < .05) one-tailed bivariate within-person correlations, as obtained in SPSS, were included in the first model. Because the relations were expected to differ between boys and girls, analyses were conducted multigroup.

In the second step, the remaining significant between- and within-person relations were added to a multigroup path model in which all relations between target behaviors, partner behaviors and later depression scores were tested. The highest CDI score of the participant in the subsequent waves was used as the outcome variable, while controlling for the initial CDI score and age of the target at wave 1. Because depression often occurs in episodes of only a few months, choosing the highest depression score was considered a better choice than a mean score over wave 2–4. To account for the fact that the highest CDI score could be in wave 2 to 4, also a control variable of the time (in months) between wave 1 and the wave of the highest depression score, was added.^3^

In the third and final step, mediation paths were tested using the model indirect command. Mediation was only tested for paths with a significant relation between the independent variable and the mediator, and between the mediator and the dependent variable.

Robust Maximum Likelihood Estimator (MLR) was used to consider non-normality of the variables.^4^ Model fit was assessed using the Comparative Fit Index (CFI) and Root Mean Square Error of Approximation (RMSEA). Good model fit is indicated by CFI’s over .95 and RMSEA’s below .05 (van de Schoot et al. 2012).

#### Retrospective Analyses

It was examined whether nonverbal behavior of adolescents that later developed (sub)clinical levels of depression differed from controls, prior to the development of (mild) depression. Also, it was examined whether partners showed different responses towards these adolescents as compared to partners of controls. Analyses were done using 2 × 2 (group x gender) Multivariate Analyses of Covariance (MANCOVA) in SPSS, with age of the target as covariate. Because of the small sample size, univariate findings were also reported when multivariate statistics just missed significance (*p* < .100).

## Results

### Predictive Analyses^5^

#### Backward Selection

Backwards selection was applied to end up with a model in which only significant within- and between-person relations remained. This resulted in a good model fit (χ^2^ (99) = 100.48, CFI = .995, RMSEA = .013) for the overall multigroup model with both girls and boys. Significant within-person correlations are displayed in Table 1. Other-oriented behaviors correlated positively with each other (except for backchannel behavior and smiling) and negatively with negative behaviors (see Table 1). So, when somebody shows a positive behavior, he or she is likely to show more positive behaviors and less likely to show negative behaviors. The only exception was the positive correlation between smiling and ‘subtle negative cues’ in boys.

**Table 1:** One-tailed bivariate within-person correlations between nonverbal behaviors for boys (*N* = 88) and Girls (*N* = 82)

	Boys					Girls				
	1	2	3	4	5	1	2	3	4	5
Target nonverbal behavior										
1. Gazing while listening										
2. Gazing while talking	.71**					.69**				
3. Smiling	−.05	−.11				.07	.16			
4. Backchanneling	.22*	.15	−.04			.00	−.00	−.27**		
5. Negative facial expression	−.46**	−.57**	.03	−.11		−.46**	−.45**	−.02	.06	
6. Subtle displeasure cues	−.18*	−.30**	.19*	−.11	.14	−.23*	−.23*	−.11	−.10	.16
Partner nonverbal behavior										
1. Gazing while listening										
2. Gazing while talking	.75**					.69**				
3. Smiling	−.15	−.09				−.05	.15			
4. Backchanneling	.12	−.04	−.19*			−.01	.04	−.29**		
5. Negative facial expression	−.46**	−.58**	.03	.01		−.45**	−.47**	.02	.15	
6. Subtle displeasure cues	−.24*	−.25**	.13	.01	.10	−.23*	−.26*	.10	−.15	.23*

Bold correlations were included in the first step of the predictive analyses

* *p* < .05, ** *p* < .01

**Fig. 1:**
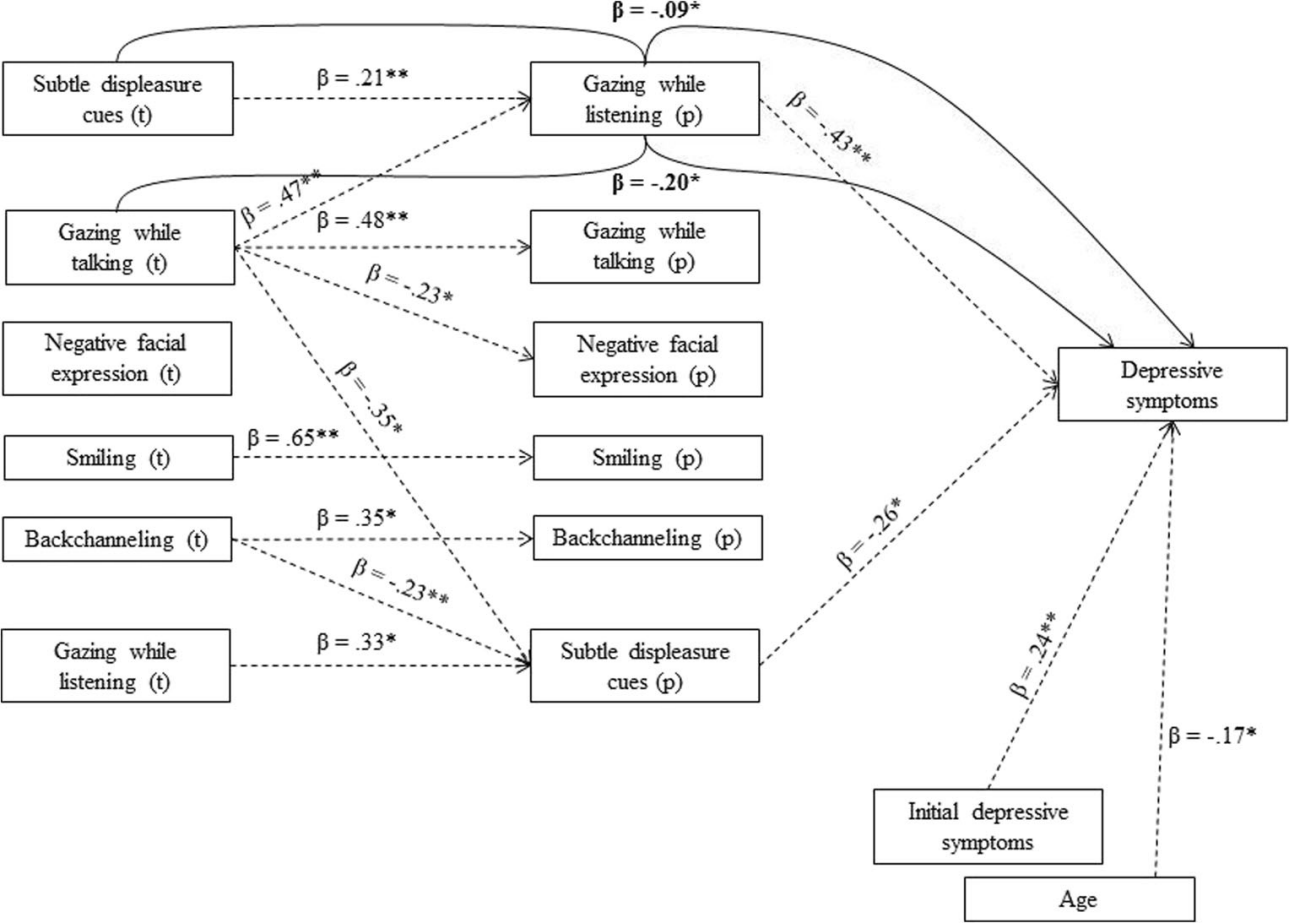
Significant regression relations for girls’ dyads (*N* = 82) from target behavior (left column) to partner behavior (middle column) and later depression of the target. Initial depressive symptoms and age of the target were controlled for. Dotted lines represent significant direct regression paths. Bold lines and statistics represent significant, indirect only, mediation paths. * *p* < .05, ** *p* < .01

**Fig. 2:**
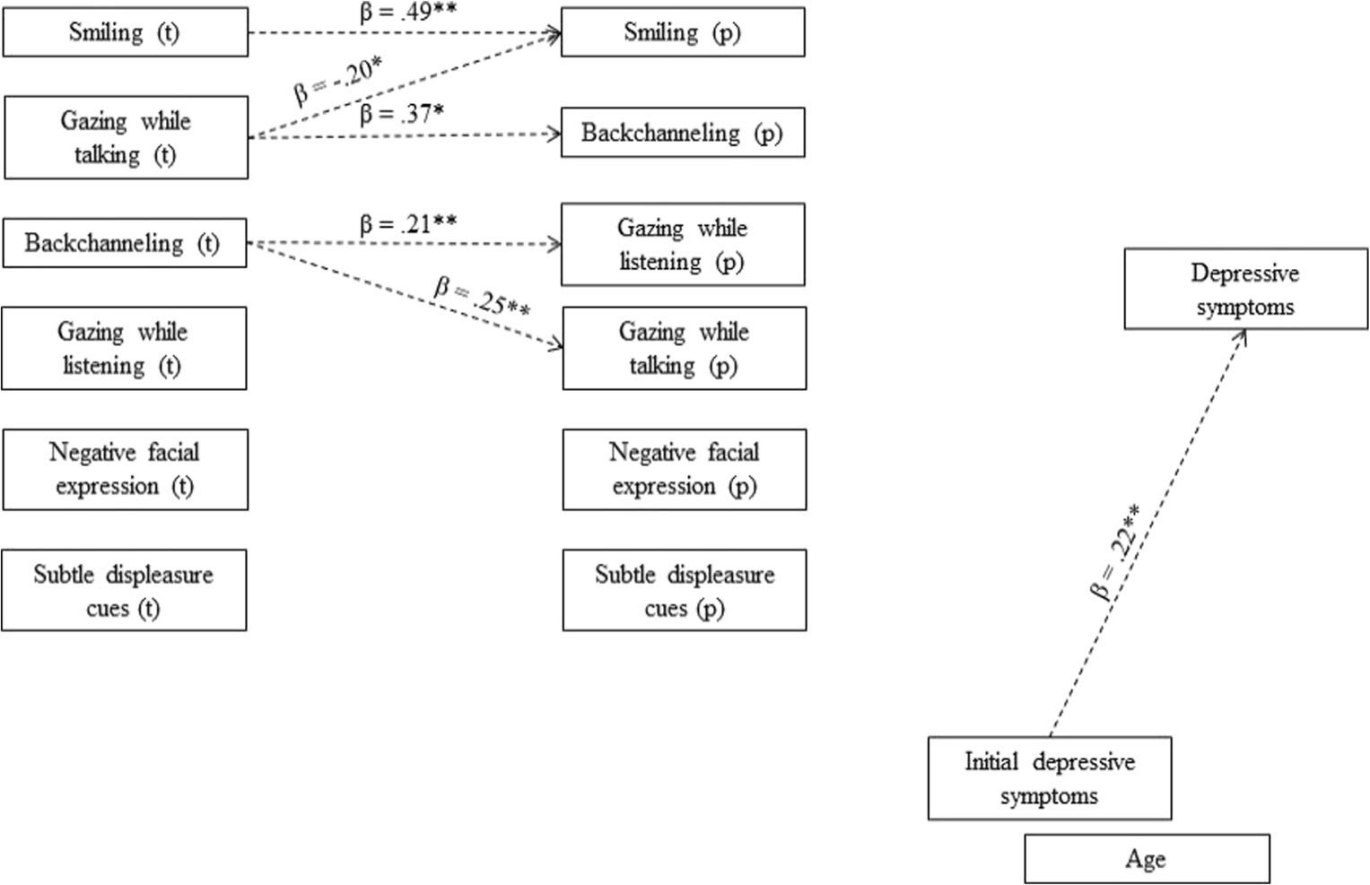
Significant regression relations for boys’ dyads (*N* = 88) from target behavior (left column) to partner behavior (middle column) and later depression of the target. Initial depressive symptoms and age of the target were controlled for. Dotted lines represent significant direct regression paths. * *p* < .05, ** *p* < .01

The significant target to partner regressions paths are displayed in the first two columns of Figs. 1 and 2 for girls’ dyads (*N* = 82) and boys’ dyads (*N* = 88) respectively. In girls, gazing while talking of target adolescents was positively related to partners’ gazing, both while listening and while talking. Smiling and backchanneling of targets were positively related to the same behaviors in partners. Thus, less other-oriented behaviors were related to less other-oriented behaviors of partners. Gazing while talking of targets was negatively related to ‘negative facial expressions’ of the partners. And gazing while talking as well as backchanneling were negatively related to partners’ ‘subtle displeasure cues’. As expected, these findings show that when targets show less other-oriented behavior, partners tend to show more negative responses. However, ‘subtle displeasure cues’ of target adolescents, contrary to expectations, was related to *more* gazing while listening in partners, and vice versa.

Different target to partner regression paths were found in boys (Fig. 2). Other oriented behaviors were not linked to the same behaviors in the partner, except for smiling. Gazing while talking of target adolescents was positively related to partners’ backchanneling, but also related to *less* partner smiling. Backchanneling of targets was positively related to both types of gazing in the partner. Negative behaviors of targets were not significantly related to nonverbal behaviors of partners, nor vice versa.

#### Predicting Depressive Symptoms

The significant within-person correlations and target to partners regression paths were included in the complete model with later depressive symptoms, resulting in the following model fit: χ^2^(171) = 165.99, CFI = 1.000, RMSEA = .000. Although chi-square and the degrees of freedom were both positive, the difference between Chi-square and df was negative, in which case the program reports a 0 for RMSEA. Although not necessarily a problem, it may indicate that the proposed SSDM model suffers from a lack of power. Because girls and boys hardly showed any overlap in the target to partner regression paths, it was decided to test two models explaining later depressive symptoms separately, one for girls and one for boys. The model for girls resulted in a good model fit (χ^2^(83) = 83.86, CFI = .995, RMSEA = .011). The model for boys again reported fit indices that may indicate lack of power and should be interpreted with caution: χ^2^(88) = 82.16, CFI = 1.000, RMSEA = .000. The model for girls explained 33.4% of the variance in depressive symptoms (*R*^*2*^ = 0.334), while the model for boys explained 18.6% of the variance in depressive symptoms (*R*^*2*^ = 0.186).

The dotted lines in Fig. 1 represent all significant direct regression paths for girls (including the already discussed target to partner behavior regression paths). No direct relations from targets’ nonverbal behavior to later depressive symptoms were found. Partners’ gazing while listening was however related to later depressive symptoms of the target, with less gazing while listening predicting more depressive symptoms. Also ‘subtle displeasure cues’ of partners were related to later depressive symptoms of the target, but contrary to expectations, this relation was *negative*. Because of the direct relations from gazing while listening and ‘subtle displeasure cues’ of partners to depressive symptoms, these partner behaviors were tested as possible mediators between target behavior and later depression (step 3). As no direct relations were found from target behaviors to depression, the tested mediation paths were ‘indirect only’ mediations, i.e. the mediator (partner behavior) does not explain the correlation between target behavior and depression, but *if* this partner behavior is correlated to the target behavior, it predicts depression. The bold lines in Fig. 1 represent the significant indirect mediation paths. Less gazing while talking of targets was related to less gazing while listening of interaction partners, which in turn predicted more depressive symptoms. More ‘subtle displeasure cues’ of targets were related to more gazing while listening of partners, in turn predicting lower levels of later depressive symptoms. The mediation path from targets’ backchanneling, via partners’ ‘subtle displeasure cues’, to depressive symptoms just missed significance (β = .06, *p* = .062).

To examine whether these results are robust, the possible mediation paths were also tested in SPSS using the Preacher and Hayes (2008) Indirect macro, thereby controlling for age and initial depression score. The results indicated that only the indirect effect from gazing while talking of targets, via gazing while listening of partners, to later depressive symptoms was significant (*B* = −.054, *SE* = .030, 95% CI = −.128 to −.007).

Fig. 2 shows the results for boys. No significant direct regression paths or indirect mediation paths were found between nonverbal behaviors to depression.

### Retrospective Analyses

To examine whether the same behavioral differences precede (sub)clinical depression, nonverbal behavior of a subsample of adolescents that later developed (mild) depression was compared to controls, prior to the increase in depressive symptoms. MANCOVA’s controlling for age revealed that adolescents that later developed (mild) depression and their partners differed in nonverbal behavior from control dyads (see Table 2). Although multivariate analysis (group x gender) for targets’ gazing behavior just missed significance (*F*(2, 56) = 2.50, *p* = .091, partial ɳ^2^ = .08), univariate findings indicated that girls that later develop depression gazed less, whereas boys that develop depression gazed more while talking as compared to controls, *F*(1, 57) = 4.96, *p* = .030, partial ɳ^2^ = .08. The same pattern for gazing while listening just missed significance, *F*(1, 57) = 3.43, *p* = .069, partial ɳ^2^ = .06.

**Table 2:** Mean levels and standard deviations gazing behaviors target adolescents and partners

	To-be depressed		Controls	
	*N*	*M (SD)*	*N*	*M (SD)*
Target boys				
Gazing while listening	14	49.24 (29.54)	14	35.16 (20.62)
Gazing while talking	14	36.39 (24.67)	14	25.12 (18.67)
Target girls				
Gazing while listening	17	52.94 (17.32)	17	60.01 (23.64)
Gazing while talking	17	28.16 (13.33)	17	39.30 (24.40)
Partner boys				
Gazing while listening	14	35.53 (22.15)	14	50.44 (32.65)
Gazing while talking	14	22.16 (17.77)	14	35.92 (31.74)
Partner girls				
Gazing while listening	17	53.76 (28.23)	17	63.28 (22.61)
Gazing while talking	17	27.08 (14.44)	17	41.98 (20.58)

In the partners, a significant multivariate main effect for group was found for gazing, *F*(2, 56) = 3.42, *p* = .040, partial ɳ^2^ = .11. Partners gazed less while talking (*F*(1, 57) = 6.78, *p* = .012, partial ɳ^2^ = .11), when interacting with an adolescent that later developed (mild) depression. The same pattern for gazing while listening was borderline significant (*F*(1, 57) = 4.00, *p* = .050, partial ɳ^2^ = .07). Group effects for other nonverbal behaviors were not significant.

## Discussion

This study examined a specific SSDM to explain the increase in depression during adolescence, especially in girls, where deviations from gender display rules were proposed as the social skill deficits. It was expected that showing less other-oriented behavior and/or more negative behavior would elicit negative reactions, particularly in girls’ dyads, which subsequently were hypothesized to predict later depressive symptoms.

### Gender Differences in Target to Partner Regression Paths

Target to partner behavior regression paths showed that less other-oriented behavior of target adolescent girls was related to less other-oriented and more negative behavior of interaction partners, as expected. There was one exception, in that ‘subtle displeasure cues’ were related to *more* gazing while listening, indicating that the partner payed extra attention to the (speech of the) target if she displayed signs of discomfort. Maybe girls try to support or reassure peers that seem to feel uncomfortable.

In boys, nonverbal behaviors of targets and partners were also related, but to a lesser extent and different than in girls. Particularly, negative behaviors of target boys were not related to less other-oriented or more negative behaviors of partners, suggesting that indeed these behaviors may not be considered violations from display rules in boys (Hall 1984). The positive within-person correlation between negative behaviors and smiling seems to further support this suggestion. As in girls, smiling was related to more of the same behavior in partners, but for gazing patterns or backchannel behaviors different regression paths were found. In boys, backchanneling and gazing while talking were mutually related, both within and between participants. As gazing while talking may indicate dominance (Dovidio and Ellyson 1982; Exline et al. 1975), it seems that this behavior is acknowledged by nodding, but not necessarily by more looking while listening. Moreover, more gazing while talking of targets was related to *less* smiling of partners, suggesting that this gazing behavior reduces the likelihood of positive emotion displays in boys, whereas these behaviors were unrelated in girls.

### Predicting Depressive Symptoms

Some evidence was found for predictive relationships from nonverbal interactions to depression, particularly in girls. Although nonverbal behavior of target girls was not directly related to later depressive symptoms, there were, indirect only, mediation paths from target behaviors, via partner behaviors, to later depressive symptoms. Gazing less while talking was related to less gazing while listening of partners, and only if this partner behavior was shown, it predicted later depressive symptoms. The retrospective study confirmed the importance of gazing in the (sub)clinical subsample. Already before the development of (mild) depression, these girls gazed less while talking than controls. Interaction partners of girls in this (sub) clinical group also displayed less gazing prior to the development of depression of the target.

Another, indirect only, mediation path was that girls’ ‘subtle displeasure cues’ were related to *more* gazing while listening of partners, which in turn was related to *lower* later depression scores. Interaction partners thus seemed to notice targets’ signs of discomfort, boredom and/or disinterest and in reaction showed more attention, possibly to reassure the other and/or make the conversation work, and if this response occurred it seemed to lower the likelihood of later depressive symptoms. However, the same mediation path was not found to be significant in post-hoc mediation analysis in SPSS, implying that the effect might not be strong. More detailed analyses of the type of displeasure cues and other co-occurring behaviors or speech content is necessary to better understand this finding.

The model in which nonverbal behaviors predicted later depressive symptoms in boys did not indicate any link between nonverbal behaviors and later depressive symptoms. Nevertheless, boys in the (sub)clinical subsample did gaze *more* while talking than healthy controls prior to the development of depression. Interaction partners of these boys in turn displayed *less* gazing (while talking), which may indicate a lack of confidence. Apparently, gazing patterns did not explain variance in depressive symptoms in the normative population but did differ in boys who later developed (sub)clinical levels of depression. It remains to be studied why high levels of gazing while talking in targets combined with lower levels of gazing in partners are seen prior to the development of (mild) depression: is the high level of gazing while talking, usually associated with confidence, regarded as too confident/dominant or does this high level of gazing often co-occur with other unfavorable behaviors (e.g., speech content) that we did not code in this study?

### Conclusion and Discussion

The present results provided limited evidence for the proposed SSDM model for depression. Only gazing patterns showed some of the hypothesized relationships between nonverbal communication and subsequent depression. Prior to (increases in) depressive symptoms girls and boys do seem to display differences in gazing behavior, as lower levels of gazing in girls and higher levels of gazing in boys were related to less gazing in peer partners and later depressive symptoms. In girls this was found both in the normative population and in the (sub)clinical group, whereas in boys this was only found in the participants developing (sub)clinical levels of depression. However, except for gazing, no other behaviors were linked to increases in depressive symptoms.

Possibly, the short duration of the observations played a role in this limitation. Gazing occurs frequently, even during 5-minute conversations, whereas the other observed behaviors occur less frequent. Future studies should use longer observations to be able to examine the relations with these less frequent behaviors more reliably. Another cause may reside in the sample size. In small samples only medium and large effects can be detected and smaller effects may be missed. Although this study is relatively large for a time-consuming observation study, it is relatively small for testing the proposed model. Larger samples are required to test this suggestion.

Also, it may be that deficits in nonverbal communication increase over time. In fact, the original SSDM (Coyne 1976a, b; Segrin 2000) proposes that before the development of depression individuals already show some deficits in behavior, but once depressive symptoms increase, behavior becomes more deviant, eliciting even more negative responses from their peers etc. Maybe it starts with different gazing patterns, while other deficits in behavior develop later. In line with this suggestion, previous studies found that girls with (mild) depression not only showed lower levels of gazing, but also more ‘subtle displeasure cues’. And they not only received less gazing, but also less smiling and more negative facial expressions from their partners (van Beek et al. 2006; van Beek and Berg 2015). In boys the picture is again less clear. Boys with (mild) depression also received less smiles and more negative facial expressions from their peers (van Beek et al. 2006; van Beek and Berg 2015), but surprisingly no differences were found in gazing or other nonverbal behaviors in the boys themselves. The higher level of gazing while talking in boys that later develop (mild) depression in the present study were not found in the (sub)clinical subsample of the earlier study. More research is required to solve this puzzle. To longitudinally test the complete SSDM for gender-specific behavior, future studies should follow adolescents, and observe their behavior from before, until during, the development of depressive symptoms, with special attention to different pathways in boys and girls.

Another open question is the cause of these ‘deviations’ in gazing behaviors. These problems may arise in adolescence due to a failure to deal with the (social) challenges in this developmental period, but may also have been present for much longer, only leading to depressive symptoms in adolescence due to cognitive and identity development. Future studies should examine the development of these gazing patterns from childhood into adolescence. This development should be studied in relation to withdrawn behavior, social or generalized anxiety as well as to personality traits such as neuroticism, as these factors may influence gazing and are known predictors for depression (Epkins and Heckler 2011; Kotov et al. 2010). Moreover, it should be studied whether the gazing patterns are predictive for depression in general or only for the common comorbidity of depression with other problems in girls such as (social) anxiety.

Also, in need of further explanation, is that no direct relations were found from targets’ nonverbal behaviors to later depressive symptoms, and thus the reported mediation paths in girls were ‘indirect only’ mediations. This implies that not all adolescent girls who displayed these ‘deficits’ in gazing showed increases in depressive symptoms. Changes in depressive symptoms seemed to occur only when certain behaviors where shown by the interaction partners. Possibly less gazing had to co-occur with other behaviors, speech content, or other characteristics of the target (e.g., attractiveness or personality traits) to elicit these partner reactions. Future studies should examine under which conditions target behaviors elicit partner responses that are related to later depressive symptoms, thereby investigating co-occurring target behaviors and other target characteristics.

Furthermore, additional sequential analyses could contribute to a better understanding of the specific links between target and partner behaviors, i.e., which (clusters of) behaviors precede or follow each other over time. Strictly speaking, the causal direction from target to partner behavior is not yet proven by the present analyses. Nevertheless, previous research in the same school population suggests that the more negative behaviors in partners may truly be responses to target behaviors, as it was found that partners who interacted with two peers, one with one without depression, showed similar differences in responses (van Beek et al. 2006; van Beek and Berg 2015).

Although participants were unaware of the focus on their behavior, future studies should examine to what extent this study’s findings regarding 5-minute interactions can be generalized to daily interactions. Since gazing patterns are important for all daily communications, we would predict that this effect may be even stronger in longer lasting, real life, interactions.

Finally, of course, additional factors may contribute to both deficits in behavior and the development of depressive symptoms. In a larger sample study, the moderating or mediating role of other known risk factors for depression should be considered, such as stressful life events, personality traits, and so forth (Epkins and Heckler 2011; Hankin 2006; Hammen 2009).

Despite the limitations, the current study was the first to show that nonverbal behaviors in targets and partners were related and could predict later depressive symptoms in adolescent girls. The similarity in findings in the normative and the (sub)clinical subsample in girls suggests that these outcomes may be clinically relevant and should be given attention in prevention and intervention programs. In boys, the picture is less clear and merits further research.

## Footnotes

1 Participants agreed that their conversations were filmed for research purposes but were initially unaware of our focus on nonverbal behavior. They were informed afterwards.

2 After correcting for age and gender bias in the CDI, Van Beek et al. (2012) indicated that boys showed similar increasing patterns in depressive symptoms, albeit that they reported slightly less symptoms than girls at all ages.

3 The effect was never significant, so it is not further discussed in the results.

4 Bootstrapping was not used since it has insufficient power for hypothesis testing in small samples (Koopman et al. 2015).

5 MANCOVA’s with age during the observation as covariate, showed that also in this study nonverbal behavior differed significantly between boys and girls, for target adolescents (*F*(6, 162) = 7.40, *p* < .001), and partners (*F*(6, 162) = 7.62, *p* < .001). As expected, girls showed more other-oriented behaviors, particularly gazing while listening, and boys showed more negative behaviors. The table with means is available from the first author.
